# Being, becoming, and belonging: reconceptualizing professional identity formation in medicine

**DOI:** 10.3389/fmed.2024.1438082

**Published:** 2024-08-27

**Authors:** Robert Sternszus, Yvonne Steinert, Saleem Razack, J. Donald Boudreau, Linda Snell, Richard L. Cruess

**Affiliations:** ^1^Department of Pediatrics and Institute of Health Sciences Education, McGill University Faculty of Medicine and Health Sciences, Montreal, QC, Canada; ^2^Department of Family Medicine and Institute of Health Sciences Education, McGill University Faculty of Medicine and Health Sciences, Montreal, QC, Canada; ^3^Department of Pediatrics and Scholar in the Centre for Health Education Scholarship, Faculty of Medicine, University of British Columbia, Vancouver, BC, Canada; ^4^Institute of Health Sciences Education, McGill University Faculty of Medicine and Health Sciences, Montreal, QC, Canada; ^5^University of Notre Dame, Sydney, NSW, Australia; ^6^Department of Medicine and Institute of Health Sciences Education, McGill University Faculty of Medicine and Health Sciences, Montreal, QC, Canada; ^7^Department of Surgery and Institute of Health Sciences Education, McGill University Faculty of Medicine and Health Sciences, Montreal, QC, Canada

**Keywords:** professional identity formation, professionalism, diversity & inclusion, socialization, education

## Abstract

Over the last decade, there has been a drive to emphasize professional identity formation in medical education. This shift has had important and positive implications for the education of physicians. However, the increasing recognition of longstanding structural inequalities within society and the profession has highlighted how conceptualizations of professional identity formation have also had unintended harmful consequences. These include experiences of identity threat and exclusion, and the promotion of norms and values that over-emphasize the preferences of culturally dominant groups. In this paper, the authors put forth a reconceptualization of the process of professional identity formation in medicine through the elaboration of 3 schematic representations. Evolutions in the understandings of professional identity formation, as described in this paper, include re-defining socialization as an active process involving critical engagement with professional norms, emphasizing the role of agency, and recognizing the importance of belonging or exclusion on one’s sense of professional self. The authors have framed their analysis as an evidence-informed educational guide with the aim of supporting the development of identities which embrace diverse ways of being, becoming, and belonging within the profession, while simultaneously upholding the standards required for the profession to meet its obligations to patients and society.

## Introduction

Over the last decade, there has been a movement in medical education to shift the focus from professionalism and professional behaviors to supporting the formation of professional identities (1). This shift has had positive implications. However, the increasing recognition of longstanding social and structural inequities within the profession and broader society have highlighted how conceptualizations of professional identity formation have also had negative and potentially harmful unintended consequences. These must be addressed.

In 2015, members of this authorship group contributed to the conceptualization of professional identity formation in medical education by publishing an article entitled “A schematic representation of the professional identity formation and socialization of medical students and residents: a guide for medical educators” (1). The schemata described in that paper synthesized the complex literature on identity and identity formation from multiple disciplines and theoretical perspectives to enable medical educators to apply it to their field (1, 2). It has become apparent that the article, along with others in the field (3–8), has had a substantial impact within the health sciences education community. The article continues to be cited frequently (with 526 citations reported as of May 21, 2024, of which 33 citations occurred between January 17 and March 25, 2024) (9), with numerous references also having been made in the literature of other academic domains. Thus, it seems reasonable to conclude that this analysis of professional identity formation continues to assist educators who wish to develop and study programs aimed at supporting professional identity formation.

While the original analysis may have been reflective of the literature at the time it was formulated, there is now compelling evidence that it does not reflect the experiences of many medical learners. This pertains to the increasing visibility of longstanding and under-recognized issues related to equity, diversity, inclusion and belonging, and their intersection with professional identity formation, as well as changes in societal norms and expectations that have impacted professional identities.

First, the formation of professional identities cannot be divorced from the socio-political context, and that context has been profoundly changed by evolutions and events over the last decade (e.g., systemic racism as exemplified by the murder of George Floyd in Minnesota and the death of Joyce Echaquan in a Quebec hospital). As a result, longstanding concerns related to equity, race, discrimination, inclusivity and oppression have been given increased voice and visibility in academic medicine and medical education. This has produced a growing body of literature that makes explicit the negative aspects of professionalism including how complexities related to professional identity formation in persons from diverse backgrounds have been neglected (10–20). We refer here to numerous commentators who have characterized professionalism as a harmful rhetorical tool to ensure conformity and uphold the traditions and values of those with the most power (i.e., the ‘weaponization’ of professionalism) (10). There is also evidence that many learners from social groups under-represented in medicine experience identity threat and exclusion (20, 21). In fact, there are increasingly expressed concerns that the establishment of professional standards, to which all are asked to adhere, have not and do not sufficiently reflect the diversity of perspectives and identities existing within medicine’s communities of practice (11, 18, 22).

Second, given that professional identities are social constructs, their nature has evolved alongside changes in societal norms and expectations. The COVID-19 pandemic (23, 24), the emergence of social media and the creation of virtual global communities of practice (23, 25, 26), increased workload demands (27), and changes to admission practices (28), among other influences, have served to further highlight inequities in health outcomes, medical practice, and medical education. This has resulted in calls for a shift in who physicians need to be to better meet the needs of society today (29).

In this paper, we propose a reconceptualization of professional identity formation in medicine through the elaboration of revised schematic representations that better reflect the current realities of medical education and practice. We describe the new schemata and the evidence that informs them, as well as how and why they have changed since 2015. We have framed the analysis as an educational guide that is based on a range of theories with different ontological and epistemological groundings. As such, this analysis is not intended to provide a theoretical perspective on professional identity formation. Rather, we intend for this evidence-informed guide to enable medical educators to better support the development of physicians whose professional identities uphold the standards required for the profession to meet its obligations to patients and society while simultaneously embracing and nurturing diverse ways of being, becoming, and belonging within the profession.

## The process of professional identity formation

To consider how medical education can support professional identity formation, the process itself must be understood (1). The process, as represented in 2015, drew on the literature at that time to describe how individuals enter medicine with unique personal identities and participate in socialization, through social engagement in the medical community, resulting in new personal and professional identities. This correctly emphasized the importance of both individuality and socialization on professional identity formation. However, recent literature has brought to light that this view of professional identity formation does not address the power dynamics that underpin professionalism, and the ways in which socialization can be experienced as a process of assimilation toward culturally dominant identities (e.g., whiteness, maleness, straightness) (13, 20, 28, 30). It has also been suggested that there may be an inadvertent implication that there is a “single aspirational and predetermined end point” to professional identity formation, and that the complete alignment or congruence between personal and professional identities is an expected result of this process. This depiction of professional identity formation does not appear to reflect the lived experiences and challenges of some medical learners. Formulating a more inclusive conceptualization will respond to an important need and may enable medical educators to better foster self-confidence, authenticity, and a sense of belonging among all learners (20, 28, 30).

**Figure 1:**
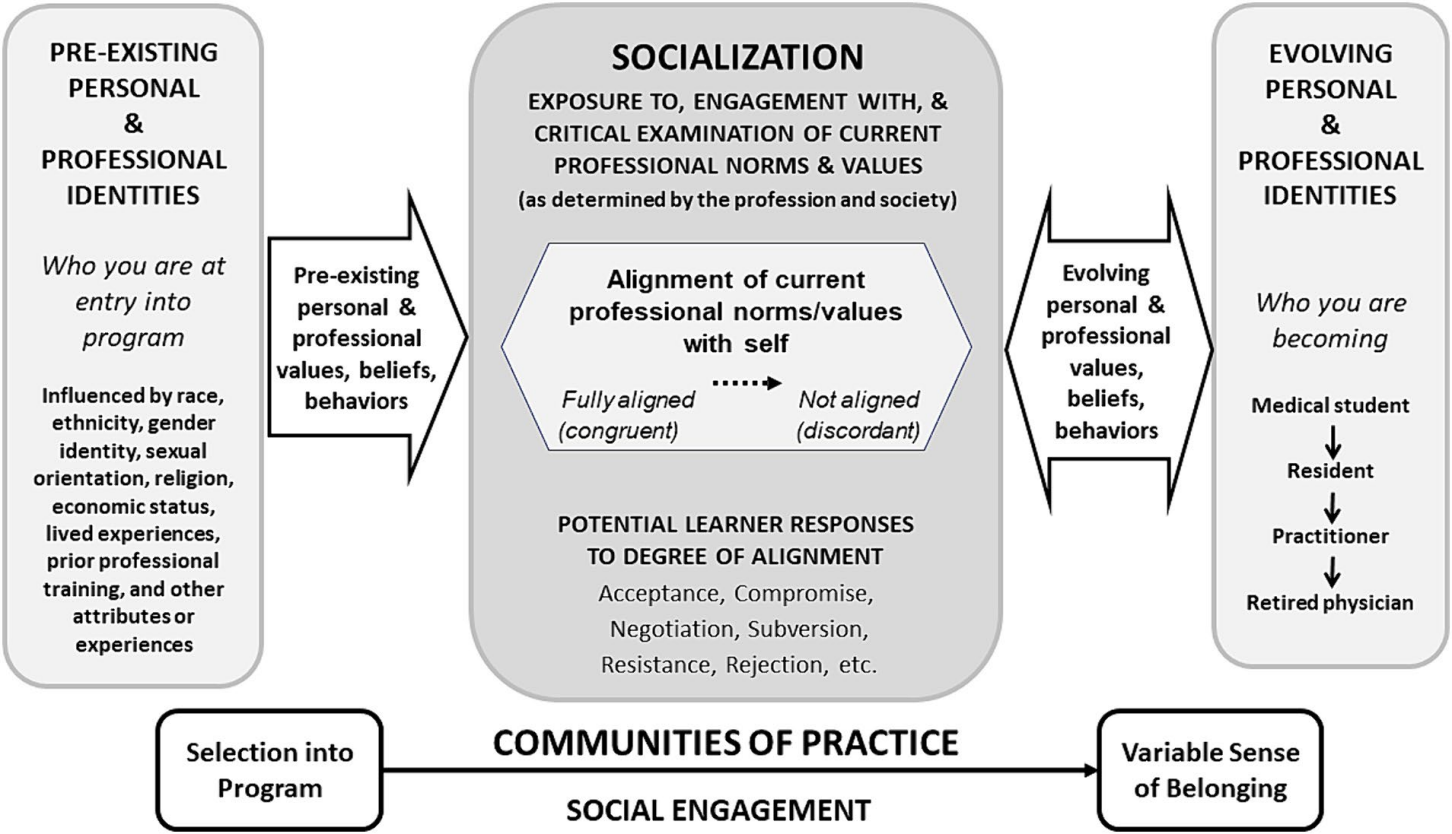
The process of professional identity formation in medicine. A revised schematic representation of professional identity formation, indicating that individuals enter the process of socialization with unique values, beliefs and behaviors that are informed by their pre-existing identities, and develop evolving personal and professional identities through a continuous process of engagement with and critical examination of the norms & values of the profession (upper portion). The process of social engagement into medicine’s communities of practice begins with selection into the program and results in learners perceiving variable degrees of belonging to those communities (lower portion). Adapted from Cruess et al. (1).

Figure 1 outlines a reconceptualized understanding of how professional identities are formed through socialization. It is divided into an ‘upper’ and ‘lower’ portion. The ‘upper portion’ has 3 components: (1) Pre-existing personal and professional identities at entry into the program; (2) Socialization and learner responses to this process; and (3) Evolving personal and professional identities. The ‘lower portion’ focuses on the trajectory of learners in communities of practice. We will elaborate in detail on each of these components.

### Pre-existing personal and professional identities at entry into the program

As in 2015, the foundational schema begins by emphasizing the individual identities that make up who learners are as they enter the socialization process. However, given that individual identities are always in evolution (31), we aim to make more explicit that ‘who you are’ as one enters the socialization process reflects identities at a discrete moment in time (i.e., at entry into a program). Further, those pre-existing identities are deeply influenced by personal and professional attributes and lived experiences. These include prior professional training, and belonging, or having belonged, to various, and potentially marginalized, social groups whose representation in medicine has implications for health equity (32, 33). Given the contentious division between ‘nature’ and ‘nurture,’ and the fact that race is a social construct (34), distinguishing between genetic and environmental influences on identity is no longer salient. Lastly, we have made explicit within the transitional arrow that ‘who you are’ is reflected in the pre-existing personal and professional attitudes, values, and behaviors brought by learners into a medical education program. That is, we have depicted professional identity formation as a process that is grounded in learners’ pre-admission identities, which are informed by a lifetime of lived experiences and attachments to various social groups. For educators, this highlights the importance of assisting learners in reflecting on, and being explicit about how their identities and backgrounds shape who they wish to be and become.

### Socialization and learner responses

The process of socialization was, and continues to be, foundational in forming a professional identity. However, the working definition of socialization that predominates in the literature, “the process by which a person learns to function within a particular society or group by internalizing its values and norms,” (35) now appears insufficient. Notably, the over-emphasis on ‘internalizing’ values and norms, which reinforces the culturally dominant ways of knowing and being, does not explicitly attend to the desired active and critical engagement of learners in the process (21, 22). Nor does it acknowledge the wide range of potential learner responses, including resistance, that can result from the degree of concordance or discordance that may be felt between the learners’ pre-existing norms and values and those of the profession (20, 27, 36–38). This original definition also did not clearly acknowledge that the norms and values of the profession are socially constructed and constantly evolving (39). The revised conception expands the scope of the socialization process to accommodate diverse ways of being and becoming (28, 30, 40).

Figure 1 depicts socialization as a dynamic and complex process that emphasizes the issues described above. First, we have stated explicitly that the norms and values that underpin socialization are dynamic (i.e., use of the word ‘current’) and are determined by the profession and by society. It is important to acknowledge that the norms in medicine’s communities of practice have typically been established by the dominant cultural group and consideration should be given to re-examining those norms through a more inclusive and constructivist process (29). Second, we emphasize not just exposure, but the importance of both engagement with, and critical examination of, professional norms. Doing so highlights the agency that learners possess and recognizes the need to examine the knowledge-power relations that underpin the medical profession. Third, because of this engagement and critical examination, we highlight that learners will experience varying degrees of alignment between their individual pre-existing values and beliefs and those current to the profession. The literature highlights that a greater degree of discordance may be felt by learners from underrepresented groups who experience important threats to their identity during the socialization process (20, 27, 36–38). Lastly, based on the degree of congruence or incongruence, learners can have a range of responses, from acceptance to rejection of professional norms and values. Learners may also subvert or suppress parts of themselves to ‘get by,’ knowing that they are adopting a way of being that may not reflect how they aspire to ‘be’ once in independent practice (38). These responses may then shape new and evolving personal and professional attitudes, values, and behaviors that will influence the identities of the learners and the profession itself.

The complexity of how experiences in the socialization process impact upon professional identity formation cannot be overstated. Explicitly creating space for learners to make sense of these experiences and grapple with moments of discordance within the socialization process is essential to supporting learners in responding to these tensions consciously and deliberately. For example, this can be done through interactional activities such as mentorship programs or group reflective exercises where learners are provided opportunities to share stories of their experiences while exploring multiple interpretations of these experiences in relation to their sense of self. These narrative exercises can enable learners to understand and claim their own evolving professional identities (7). In turn, they can help foster authenticity and a sense of inclusion in the process of forming a professional identity (28, 30).

### Evolving personal and professional identities

As in 2015, the revised schematic representation highlights that personal and professional identities form through the socialization process and, that different identities form as one moves through transitions in training (e.g., from pre-clinical medical student to clinical clerk or intern, from resident to practitioner) (1). In addition, Figure 1 emphasizes the constantly evolving nature of both personal and professional identities. Like personal identities, professional identities change throughout one’s career and life, up to and during retirement (41–43). Physicians continuously experience socialization and exert their own influence upon the profession (44), as indicated by the double-sided arrow on the right-hand side of the schema. Further, while personal and professional identities develop in congruence, many learners and physicians report engaging in behaviors whereby their professional identity reflects a persona that is not authentic to their personal sense of self (45). Evolving ‘personal identities’ and ‘professional identities’ are deliberately kept distinct in Figure 1 to reflect this reality. For educators, this highlights the importance of empowering learners to recognize their ability to exert influence on the profession (e.g., having them reflect on changes to the norms of practice like work hour limitations or parental leave, that have been driven by learners). Educators can support authenticity in the face of adversity and help learners continue to strive to become who they intend to be, working to make the profession a more inclusive and representative institution.

### Communities of practice

The ‘lower portion’ of this revised schema charts the path of a learner from admission into the program toward a variable sense of belonging, through a process of social engagement, into various communities of practice. This portion of the schema has also changed in important ways since 2015.

First, the term ‘Community of Practice’ has been replaced with ‘Communities of Practice.’ This change reflects a shift in the theory of *Communities of Practice* (46), to a theory of *Landscapes of Practice* (47), which emphasizes that social learning occurs in a network of interconnected communities. For example, as one becomes a medical student, one joins a community of physicians, a faculty of medicine, a university, a class, and occasionally local groups or clubs. Social engagement in these communities impacts professional identities. Second, we have changed the language on the two ends of the scale. Rather than staying true to the language of ‘legitimate peripheral participation,’ and ‘full participation’ used in Lave and Wenger’s original theory (46), we aim to be descriptive of the process and cognizant of power dynamics that are at play. The word ‘legitimate’ is problematic in this context and it is not immediately clear how legitimacy is determined. Given that what we are referring to here is, and has always been, selection into a program, it is now stated in this way. In addition, while all who complete training and acquire a license to practice medicine can achieve ‘full participation’ in the practice of medicine, not everyone feels that they can fully participate in, or belong to, a given community of practice (28, 45). The explicit emphasis on belonging highlights the need for educators to attend not just to full participation in medical acts but also to meaningful inclusion into communities of practice through valuing and recognizing the individuality and diverse perspectives of their learners (40, 48).

### Summary of the process of professional identity formation

Figure 1 conceptualizes socialization as a series of processes whereby individuals develop new and evolving personal and professional identities through social engagement in communities of practice. It emphasizes the social nature of learning and the integration of professional norms and values. By highlighting diverse pre-existing identities, the importance of critical examination, the role of learner agency, and the importance of belonging, this revised conceptualization of professional identity formation provides educators with a framework in which to foster individuality, diversity, and the development of authentic professional identities. In turn, conceptualizing professional identity formation in this way can have positive implications for learner well-being and health equity (30, 33, 40, 48).

## Factors influencing the socialization process

**Figure 2:**
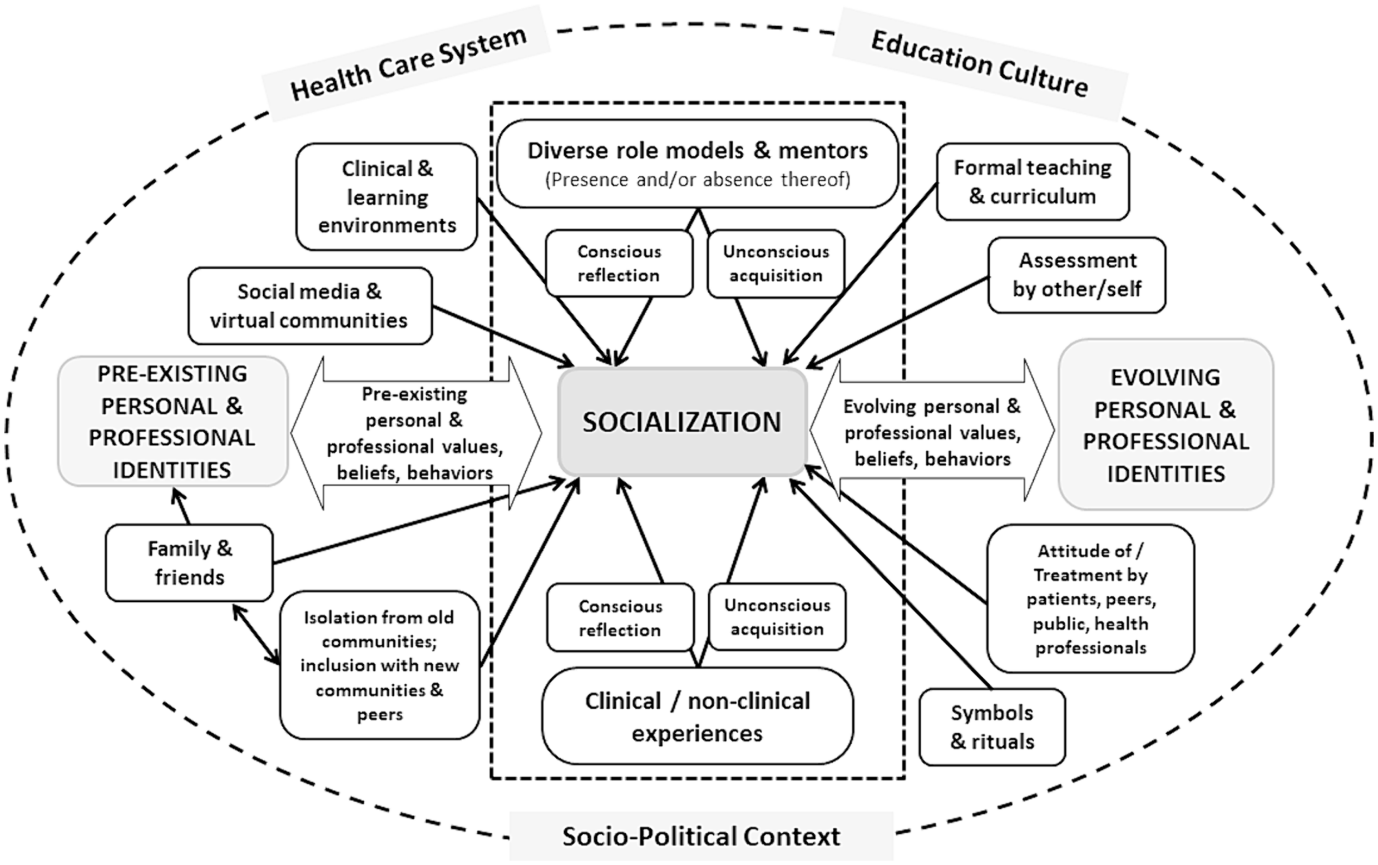
Factors influencing socialization into medicine’s communities of practice. A revised schematic representation of the multiple factors involved in the process of socialization in medicine upon which medical education can impact. The large center box surrounded by the dotted line, which includes diverse role models and mentors and experiential learning, indicates their importance to this process. The large circle surrounding the entire figure highlights the importance of the real world (including its sociopolitical context), the culture of the education system and the impact of the healthcare system within which individuals must both learn and practice, on the process of socialization. Adapted from Cruess et al. (1).

The complex set of factors influencing the socialization process is illustrated in Figure 2. Each box in the figure represents a factor (e.g., clinical experiences, formal curriculum, learning environments) whose impact can be mediated through the educational process. Many of the factors depicted remain as relevant today as they were in 2015. However, we believe that 3 major revisions are necessary to better reflect current understandings of professional identity formation.

First, it is important to be explicit that the process of socialization does not take place in isolation from the real world (including its sociopolitical context) or the culture of the education system and is not immune from the impacts of the healthcare system within which individuals must learn and practice. It has long been recognized that there are profound differences in the attitudes, values, and practices accepted as the norms in different countries and cultures (49–52). Even among cultures that appear to be similar, there can be significant differences in the nature of the social contract between medicine and society resulting in differences in the professional identities that emerge (53). One needs only to look at publicly-funded health care in countries like Canada and the more market-oriented approach in the United States to appreciate the validity of this statement (54). In addition, the culture of educational institutions can vary widely, as manifested in their vision, mission statements, and educational objectives; these can have a profound impact on the identity of graduates (55). For example, the career paths of graduates of large urban medical schools and universities, in contrast with those whose education has taken place in smaller rural communities, may differ vastly (56). Moreover, as the structure, organization, and funding of healthcare systems throughout the world have evolved, it has become apparent that professional identities are in part dictated by the nature of the environment within which individuals must practice. Hafferty and Castellani documented the presence of multiple forms of professionalism in contemporary society (57). For example, more individualistically framed and altruism-centered approaches as highlighted in US-based literature, and more public-centric and social policy-oriented approaches emphasized in European-based writings, promote different professional identities (57). Therefore, Figure 2 is enclosed by a circle to highlight the above statements and encourage educators to help learners consider the impacts of these structures and contexts on their evolving sense of professional self.

Second, social media and virtual communities have been explicitly included as a factor that influence socialization and professional identity formation (26). Virtual platforms are increasingly used for educating, communicating professional values, sharing knowledge, bringing together diverse health professionals and providing support to learners (23, 25, 26, 58). The widespread use of social media has also led to a blurring of lines between personal and professional identities. At times, this has challenged learners, institutions and regulatory bodies alike (23). Given the potential of social media and virtual communities to influence professional identity formation, both positively and negatively, formally highlighting them as an opportunity for educational interventions in support of professional identity formation is worthwhile.

Third, in the original (2015) article, an assumption was made that role models and mentors would be plentiful and that, while they could exert a negative impact, their influence would be largely positive (59, 60). There is now an extensive literature which documents that role models can, unfortunately, have negative impacts (38, 40, 44, 61–65). Of even more importance is the absence of models whose conduct can serve as a guide for underrepresented and marginalized groups in the medical profession (10, 12, 65). The paucity of females in academic leadership positions (63), as well as the lack of individuals from indigenous communities and racial minorities in medicine (64, 65), imposes extraordinary burdens on those who lack a familiar figure as a role model. It also changes the nature of the community, significantly diminishing the sense of belonging that is so fundamental to being and becoming a doctor (12). Figure 2 reflects this important reality by the inclusion of the factor entitled ‘diverse role models and mentors.’ For educators, this highlights the need to explicitly attend to supporting learners in identifying diverse role models that can reflect the many facets of their identities.

## Learner experiences and reactions to those experiences

**Figure 3:**
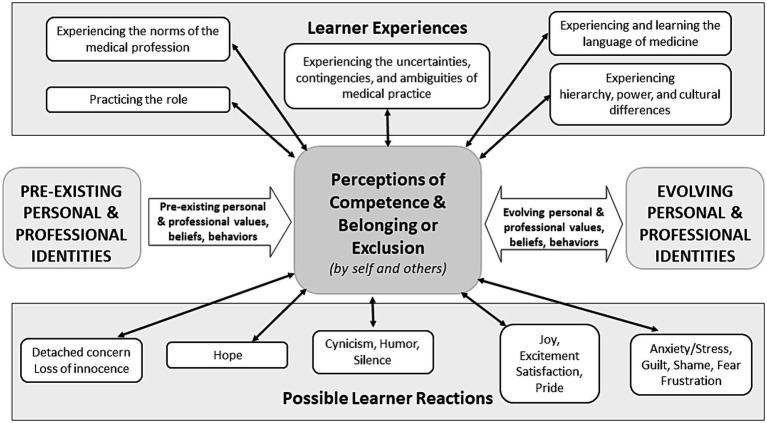
Learner experiences and reactions during the process of socialization. A revised schematic representation of the common experiences of learners (upper portion) and frequently expressed reactions and emotions in relation to those experiences (lower portion) during the socialization process. These experiences and associated reactions significantly impact upon perceptions of competence and belonging or exclusion, as determined by self and by others. In turn, these have an important influence on evolving personal and professional identities and their formation. Adapted from Cruess et al. (1).

As learners engage in the various experiences of medical education, the impact of emotions, self-conscious emotions, and emotional intelligence on professional identity formation is significant (66–69). To capture this, Figure 3 has two sections. The ‘upper portion’ depicts the common experiences of learners during the process of socialization (e.g., experiencing hierarchy, practicing the role of physician); the ‘lower portion’ documents frequently expressed positive (e.g., joy, excitement) and negative (e.g., anxiety, shame, fear) emotions as well as varied possible reactions (e.g., detached concern, hope, humor, silence) in relation to those experiences (66–72). In turn, we depict how both the experiences and the associated emotions and responses influence perceptions of competence and belonging, which are central to the process of socialization.

In the original paper, we referred to ‘increasing competence’ as being vital to one’s sense of professional identity. This view was rooted in historical notions of competence as the progressive attainment of the minimum level required for the practice of medicine (73). However, our understanding of the nature of competence in medicine has become increasingly complex. The trajectory toward proficiency is far from linear, and perceived competence can vary greatly across contexts or more personalized domains of expertise (73). Furthermore, one’s sense of competence is reflected both in how one perceives oneself and how one is perceived by others (22). Therefore, Figure 3 highlights the central role of perceptions of competence, as determined by self and others, on the socialization process. For example, the identity of a senior skilled clinician is profoundly different from that of a recent graduate. This will be reflected in two important dimensions: in the respect given to senior members of the profession by their peers and by the level of confidence exhibited by the individual (74). Recognized experts can speak with authority and the profession’s internal reward systems often contribute to this authority (3, 4, 74). This, in turn, impacts professional identity and its formation.

In addition, the importance of feeling a sense of belonging and/or exclusion on professional identity formation has featured prominently in recent literature (20, 27, 40, 48). Perceptions of belonging, by the self and by others can be importantly affected by experiences that impact upon the ability of a learner to recognize themselves within the profession (20, 27). As a result, Figure 3 highlights the central role of perceptions of belonging, alongside perceptions of competence, in the formation of a professional identity.

The orientation of schema 3 around the role of learner experiences, emotions and reactions in shaping perceptions of competence and belonging by self and others has important implications for educators. Helping learners to recognize and reflect upon their emotions and internal reactions as well as explicitly attending to the experiences that tend to produce said reactions (e.g., through narrative story-telling exercises) can provide an important catalyst for growth and development for the learner as well as the profession.

## Conclusion

The conceptualization of professional identity formation, as depicted in the schematic representations originally published in 2015 (1), continue to serve prominently in education research and practice. While this has positively served the community in many ways, this understanding of professional identity formation appears to not fully reflect the current reality of those engaging in socialization in medicine’s communities of practice. This is the result of significant changes in global socio-political contexts, evolution of relevant theories, and an increased recognition of the need to support diverse learners’ professional identity formation for authentic and meaningful practice. Authentic practice is directly linked to health outcomes, which alone provides a justification for review and change. Further, professionalism and professional identity formation are dynamic and evolving social constructs (39, 75, 76), and as such, our understanding of professionalism and professional identity formation must evolve in parallel with changing societal expectations and norms. It is therefore almost certain that further updates will be needed as we continue to learn and grow as physicians, people, and societies. That said, the authors hope that this reconceptualization of professional identity formation can serve to better guide educational interventions aimed at ensuring that those emerging from health sciences education systems have developed professional identities capable of delivering competent, compassionate, and equitable healthcare and meeting diverse societal needs. Further, it can direct educators in how to support professional identity formation in ways that enable all individuals to maintain the essential and diverse aspects of who they are while also being empowered to contribute to the continued development and flourishing of the profession. Finally, it is hoped that the process of being and becoming a physician is accompanied by a profound sense of belonging to welcoming and supportive communities of practice whose inspirations, aspirations, rules, governance, hierarchy, power relationships, values, and attitudes have been shaped by representative community members and are therefore acceptable to and reflective of all.

## Previous presentations

Components of the 3 schematic representations (not in the exact form they appear in the manuscript) were presented at the International Conference on Residency Education (ICRE), October 19–212,023, Halifax, Nova Scotia, Canada.
